# Neurogenesis decreases in the offspring of mothers infected with influenza A virus

**DOI:** 10.3389/fcimb.2025.1704546

**Published:** 2026-01-06

**Authors:** Anastasiya Rakovskaya, Alexey Lozhkov, Yana Zabrodskaya, Valeria Kirenskaya, Olesya Korovina, Angelika Garshinina, Valeria Zryacheva, Anna Shtro, Ekaterina Pchitskaya, Olga Vlasova, Ekaterina Elpaeva, Dmitry Moshkoff, Maria Salvato, Ilya Bezprozvanny, Andrey Vasin

**Affiliations:** 1 Institute of Biomedical Systems and Biotechnology, Peter the Great St. Petersburg Polytechnic University, St. Petersburg, Russia; 2 Smorodintsev Research Institute of Influenza, St. Petersburg, Russia; 3 The Global Virus Network (GVN) Center of Excellence at Institute of Biomedical Systems and Biotechnology, St. Petersburg, Russia; 4 Department of Veterinary Medicine, University of Maryland, College Park, MD, United States; 5 Laboratory of Molecular Neurobiology, Pavlov Institute of Physiology Russian Academy of Sciences, St. Petersburg, Russia

**Keywords:** influenza virus, neurodevelopment, neurogenesis, neuroinflammation, offspring, pregnancy

## Abstract

**Introduction:**

Seasonal influenza virus infection during pregnancy poses significant risks to maternal and fetal health, contributing to adverse neurodevelopmental outcomes in offspring. This study investigates the impact of maternal infection with two highly pathogenic H1N1 influenza A virus (IAV) strains on hippocampal neurogenesis and glial reactivity in neonatal and juvenile mice.

**Methods:**

Mice were infected with the mouse-adapted influenza virus strain A/WSN/33 (H1N1) or A/California/07/09 (H1N1)pdm09 in a sublethal dose 14 days after pregnancy manifestation. After birth, several pups were sacrificed, brains and hippocampi were isolated and used for RT-qPCR (the expression of IL-1β, iNOS, IFNG, IL-6, TNFa was assessed), immunohistochemistry and Western blot for markers of neural progenitors (Sox2, Sox11), mature neurons (NeuN), microglia (Iba1), and astrocytes (GFAP). Within 2 weeks after birth, the mortality and body weight dynamics change were monitored in the remaining pups.

**Results and discussion:**

Findings reveal that maternal infection with H1N1wsn disrupts early neurogenesis, while infection with H1N1pdm09 induces region-specific reductions in neurogenesis and heightened glial reactivity in 14-day-old offspring. Increased expression of pro-inflammatory cytokines and factors, including IL-1β and iNOS, in neonatal brain tissue suggests that maternal immune activation mediates neurodevelopmental disruptions. Despite reduced Sox2+ and Sox11+ neural progenitor cells, NeuN expression remained stable, implying potential compensatory mechanisms. Elevated astrocyte reactivity in the CA1 and dentate gyrus regions highlights prolonged neuroinflammatory effects. These results underscore the role of maternal influenza-induced immune responses in shaping hippocampal development, with implications for long-term cognitive and behavioral outcomes. Understanding these mechanisms may inform strategies to mitigate neurodevelopmental risks associated with prenatal infections.

## 1 Introduction

Seasonal influenza virus causes 3–5 million severe cases worldwide. Influenza infection is one of the major risk factors during pregnancy. During the H1N1 pandemic in 2009 it was noted that a severe disease course can cause preterm birth or low birth weight (Newsome et al., 2019). Maternal influenza infection leads to serious morbidity and mortality in pregnant women, as well as increases the risk of adverse fetal outcomes (preterm birth, low birth weight, high risk of infant mortality, impaired respiratory mucosal immunity, and neurodevelopmental disorders) (Liong et al., 2023; Egorova et al., 2024). It has been reported that prenatal influenza infection can be associated with the risk of developing schizophrenia (Byrne et al., 2007; Alvarez-Mon et al., 2021), psychosis or psychosis-like experiences (Zammit et al., 2009; Dreier et al., 2018), mood disorders (Morgan et al., 1997), developmental delays (Morgan et al., 1997), and bipolar disorder/bipolar affective disorder with psychotic features (Canetta et al., 2014).

Excessive activation of the maternal immune system in response to infection is one of the major causes of cytokine induction in the placenta and neurodevelopmental disorders in the offspring (Tsukada et al., 2019; Xu et al., 2024). In mice models, no viral RNA has been found in the placenta or fetal tissues (Chan et al., 2010). However, influenza-induced complications can occur without vertical transmission, including necrosis and apoptosis of placental trophoblast, pulmonary congestion and myocardial degeneration in the fetus, fetal liver necrosis (Chan et al., 2010). Several pathologies in the offspring such us developmental delay, neuropsychiatric disorders may be also associated with influenza-induced maternal inflammation (Fuentes-Zacarías et al., 2021). It was shown earlier (Jurgens et al., 2012), that proinflammatory cytokines (IL-1β, IL-6, TNF-α, IFN-α) and microglial reactivity were increased, while neurotrophic (BDNF, NGF) and immunomodulatory (CD200, CX3CL1) factors were decreased in the hippocampus of influenza infected adult mice. In our work we evaluated neuroinflammation and neurodevelopment of newborn pups after maternal influenza virus infection, which allows us to evaluate the initial mechanisms of the influence of maternal immunity activation on the offspring neurological disorders.

This way, although influenza virus usually cannot penetrate through the placenta, it can induce immune activation and inflammation in placental tissues. Specifically, interferons (IFN), interleukin-1β (IL-1β), IL-6, IL-17A and tumor necrosis factor-α (TNF-α) are elevated in the maternal bloodstream (Brown, 2006; Marcelin et al., 2011; Choi et al., 2016; Rudolph et al., 2018; Elgueta et al., 2022). Maternal cytokines are able to cross the placental barrier and induce a prolonged inflammatory response in the fetus. Moreover, the cytokines can penetrate through the immature blood–brain barrier and play a key role in disruption of neurodevelopmental processes in the brain (Novak et al., 2019; Tsukada et al., 2019; Xu et al., 2024). For instance, IL-6 and IL-17A act together and cause cortical malformations associated with ASD behavioral abnormalities (Estes and McAllister, 2016; Tsukada et al., 2019). IL-6 overexpression attenuates neurogenesis in the hippocampal dentate gyrus (Vallières et al., 2002). Proinflammatory IFN-γ-mediated signaling can induce iNOS expression, NO release to a critical neurotoxic level and neurodegeneration (Papageorgiou et al., 2016). Also, IFN-γ stimulates the production of TNF-α, superoxide radicals and interacts with GluR1 subunit of AMPA receptor in neurons that increases Ca^2+^ influx and NO production (Mizuno et al., 2008). IL-1ß is implicated in microglia activation (Eloundou et al., 2019), pathophysiological processes in CNS and NMDA-mediated neurotoxicity (Viviani et al., 2003). Blocking of IL-1β signaling protected developing mice from perinatal brain injury (Tsimis et al., 2017). The cytokine is also a potent inducer of iNOS expression (Licinio et al., 1999). This way, prenatal infection and inflammation can cause direct injury to neurons and neural progenitor cells or indirect injury through activation of microglia and astrocytes, which can trigger cytokine and inflammatory factors expression and oxidative stress in brain tissues (al-Haddad et al., 2019).

Maternal immunity activation is reported to be the major cause of neurodevelopmental disorders in offspring. Increased level of IL-6, IL-1β, TNFα, and IFN-β in response to influenza can damage the placenta and cause intrauterine fetal growth impairments (Xu et al., 2024). IL-6 in combination with TGF-β promotes the differentiation of maternal naive T cells towards helper T cells with a Th17 phenotype that can cross the placenta and produce IL-17A. Infection of pregnant mice has been linked to alterations in IL-17A, which lead to fetal immunity activation and neurodevelopmental problems (Elgueta et al., 2022). Cytokines IL-17A, IL-6 and TNFα, may reach the placenta, where they additionally activate resident immune cells, resulting in an increased production of proinflammatory cytokines. Moreover, activated maternal Th17 cells also transmigrate through the placenta and enhance cytokine production, which affects placenta function and causes damage. This allows the cytokines to activate fetal immunity and induce inflammation. Then, proinflammatory cytokines cross the developing blood-brain barrier and initiate neuroinflammation.

The hippocampus, a critical region for learning and memory, is particularly vulnerable to inflammation and other stressors during development (Leuner and Gould, 2010; Eisch and Petrik, 2012). Neurogenesis, the process of generating new neurons from neural stem and progenitor cells, is highly active in the developing hippocampus and plays a pivotal role in its functional maturation (Aimone et al., 2014; Kempermann et al., 2015). Disruptions to neurogenesis during critical developmental windows may have lasting consequences for brain function and behavior (Zhao et al., 2008; Nelson and Gabard-Durnam, 2020). Previous studies have implicated maternal viral infections in impairing neurogenesis, potentially through the induction of neuroinflammation (Jurgens et al., 2012; Antonson et al., 2019), but detailed insights into these processes remain scarce. Neuroinflammation, characterized by elevated levels of pro-inflammatory cytokines, activation of glial cells such as microglia and astrocytes, and infiltration of peripheral immune cells, is a hallmark response to viral infection in the CNS (Ransohoff and Brown, 2012; Heneka et al., 2014; Woodburn et al., 2021). While this response aims to eliminate pathogens and restore homeostasis, it can also lead to collateral damage, including neuronal injury and altered neural circuitry (Block et al., 2007; Harry and Kraft, 2012). Microglia, the brain’s resident immune cells, play a central role in responding to infection through activation and phagocytosis (Kettenmann et al., 2011; Wolf et al., 2017), while astrocytes modulate immune responses and support neuronal survival (Wilhelmsson et al., 2006; Colombo and Farina, 2016). Dysregulation of these glial responses during development may contribute to long-term impairments in hippocampal structure and function (Sofroniew and Vinters, 2010; Bolton et al., 2022).

In this study we investigated the impact of maternal influenza virus infections with highly pathogenic H1N1 influenza virus strains on hippocampal neurogenesis and glial reactivity in offspring during early postnatal development. We used two mouse-adapted strains: A/WSN/33 (H1N1), herein referred to as H1N1wsn and A/California/7/09 (H1N1), referred to as H1N1pdm09. The findings highlight that infection with H1N1wsn disrupts early neurogenesis in neonatal pups (P0), while H1N1pdm09 infection triggers region-specific reductions in neurogenesis and heightened glial reactivity in 14-day-old mice. These results provide insight into the differential effects of maternal influenza infections on brain development, emphasizing the importance of addressing virus-induced neuroinflammation as a potential strategy to mitigate long-term neurological consequences in offspring. This research provides new insights into how maternal viral infections influence hippocampal development and highlights the potential long-term consequences of neuroinflammation during critical periods of brain maturation.

## 2 Results

### 2.1 The survival rate of pups is dramatically reduced by H1N1 infection

**Figure 1:**
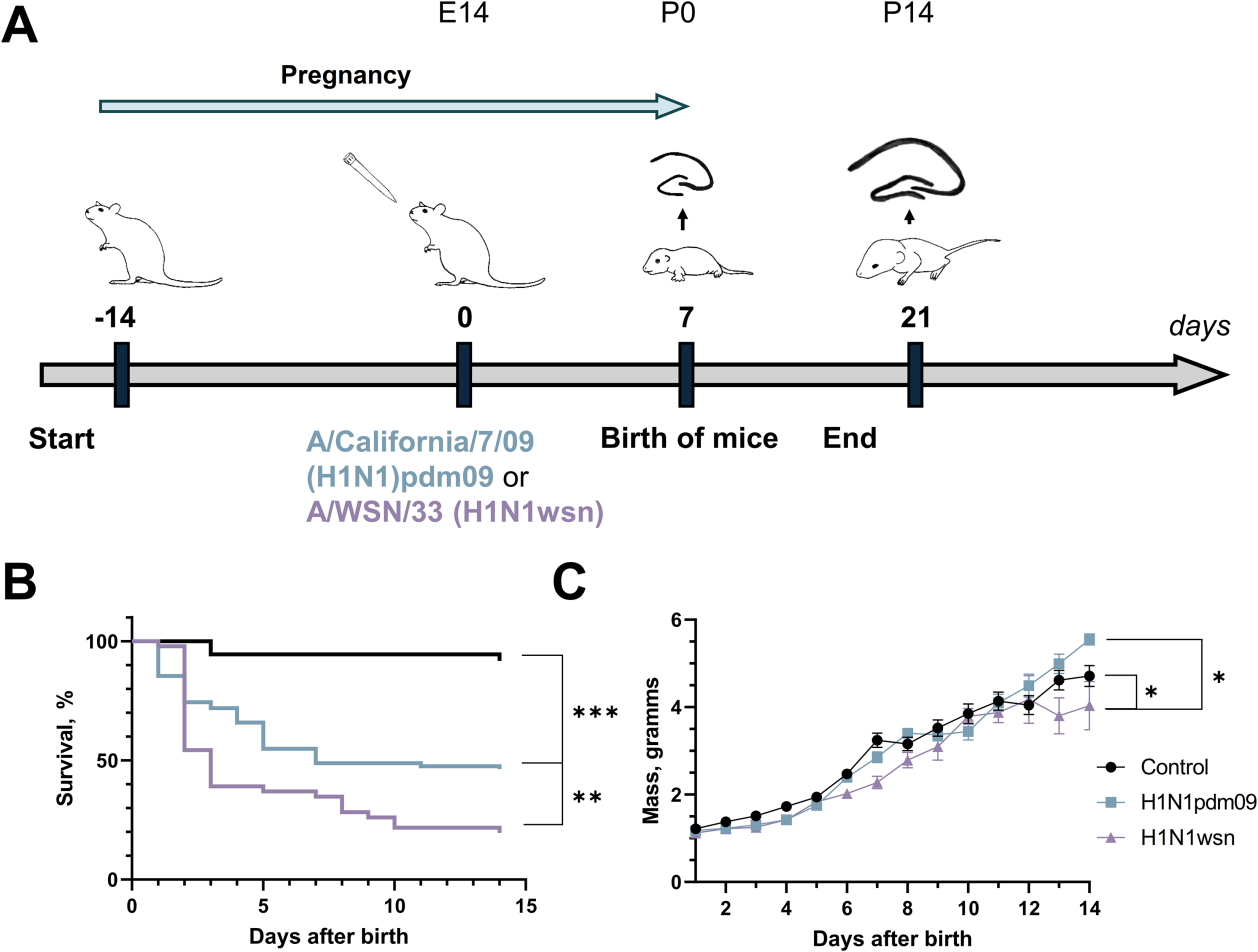
Infection with influenza virus resulted in high mortality and morbidity in the offspring. **(A)** The scheme of the experiment. **(B)** H1N1pdm09 (N = 79, from 18 litters; blue) and H1N1wsn (N = 43, from 11 litters; lilac) reduced the survival rate of newborn pups compared with mock infected control (N = 36, from 8 litters; black). Survival of H1N1pdm09 pups was significantly better than survival of H1N1wsn pups. Data are presented as Kaplan–Meier survival curves. **p < 0.01, ***p < 0.001, statistical analysis was performed using the Mantel-Cox test, with Bonferroni correction for multiple comparation. **(C)** Dynamics of body weight changes in pups whose mothers were infected with H1N1pdm09 (blue, square) and H1N1wsn (lilac, triangle). In the case of H1N1wsn, pup’s weight was decreased compared with control and H1N1pdm09. Data are presented as mean ± SEM. *p< 0.05, statistical analysis was performed using repeated measures one-way ANOVA with Tukey’s multiple comparisons test.

In total, 33 pregnant mice were infected with H1N1pdm09, 24 with H1N1wsn in a sublethal dose at about 14 days after pregnancy manifestation. In mock infected group (mice, infected with PBS instead virus), 15 pregnant females were included. The detailed scheme of the experiment is shown in **Figure 1A**. On the first day after birth, several pups were sacrificed, brains and hippocampi were isolated and used for RT-qPCR (2 or 3 pups from the litter), immunohistochemistry and Western blot (4 pups from the litter). Within 2 weeks after birth, the mortality and body weight dynamics change were monitored in the remaining pups.

Among pregnant mice infected with H1N1wsn, significant mortality after childbirth was noted (survival rate was about 47%). In the case of H1N1pdm09, the survival rate of mothers was 68%. Only 1 out of 15 mothers died after childbirth in the control group (**Supplementary Figure S1**). Viral RNA was detected in lung homogenates after childbirth. Moreover, the expression of *Oas1a/g*, *Irf7*, and *Il6*, that are involved in the immune response to influenza infection, was elevated in lungs (**Supplementary Figure S2**). We did not detect the presence of viral RNA in brain tissues of newborn pups (**Supplementary Figure S3**), indicating that neurodegenerative disorders in the offspring are the consequence of maternal immune system activation rather than direct penetration of influenza into the brain.

Maternal IAV infection also resulted in significant mortality in the offspring (**Figure 1B**). In mock infected group, the litter size varied widely, from four to twelve neonates. We noted a trend towards a decrease in the number of newborn pups in infected dams, however the differences were on the verge of statistical error (**Supplementary Figure S4**). It should be noted that several infected mothers were prone to devouring their newborn pups, which complicates the assessment of the number of viable neonates. In the case of maternal H1N1wsn infection, neonates were noted to have reduced weight (**Supplementary Figure S5**).

The survival rate in the control group was about 92%, in the H1N1pdm09-infected group it was approximately 46%, and in the H1N1wsn group no more than 20% of newborn mice survived. Significant differences in survival curves were shown both in the case of infection with different IAV strains (H1N1pdm09 and H1N1wsn) and with mock infected control. One of the possible explanations of the results is maternal morbidity. Influenza-infected postpartum dams were characterized by reduced ability to perform appropriate maternal care. However, cross-fostering pups from infected to control dams often led to the loss of the pups, therefore, we decided to avoid this practice. This way, in our experimental model, pups’ loss could be partly contributed to influenza-induced pregnancy complications such as abandonment, hypothermia, suboptimal nutrition.

Also, mother’s infection with H1N1 led to weight loss in the growing offspring. The dynamics of body weight changes in pups are shown in **Figure 1C**. These data indicate that infection of pregnant C57BL/6 mice with H1N1pdm09 and H1N1wsn was associated with significant morbidity and mortality in the offspring.

### 2.2 Assessment of cytokines, inflammatory and neurotropic factors expressed in the brain, and hippocampus of newborn mice

**Figure 2:**
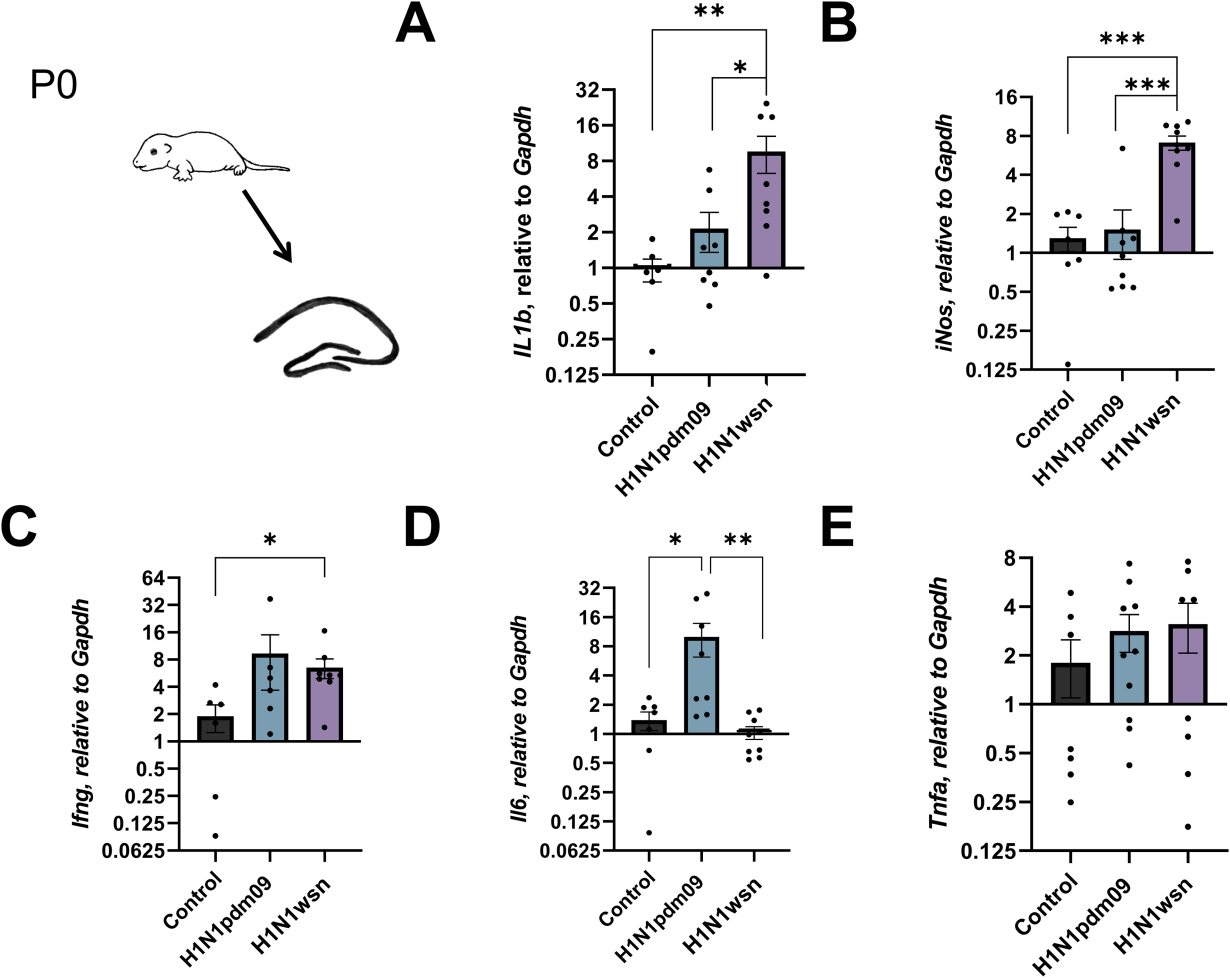
Analysis of mRNA expression of **(A)***Il1b*, **(B)***iNos*, **(C)***Ifng*, **(D)***Il6*, **(E)***Tnfa* in the hippocampi of newborn pups (p0) whose mothers were infected with H1N1pdm09 or H1N1wsn. The offspring of influenza infected pregnant mice was used for the genes expression assessment; 4 independent litters; H1N1pdm09–10 pups, H1N1wsn – 11. Mock infected group is consists of 3 independent litters, 8 pups. Data are presented as mean ± SD. *p < 0.05; **p<0.01; ***p<0.001. Statistical analysis was performed using one-way ANOVA with Tukey’s test for multiple comparisons.

**Figure 3:**
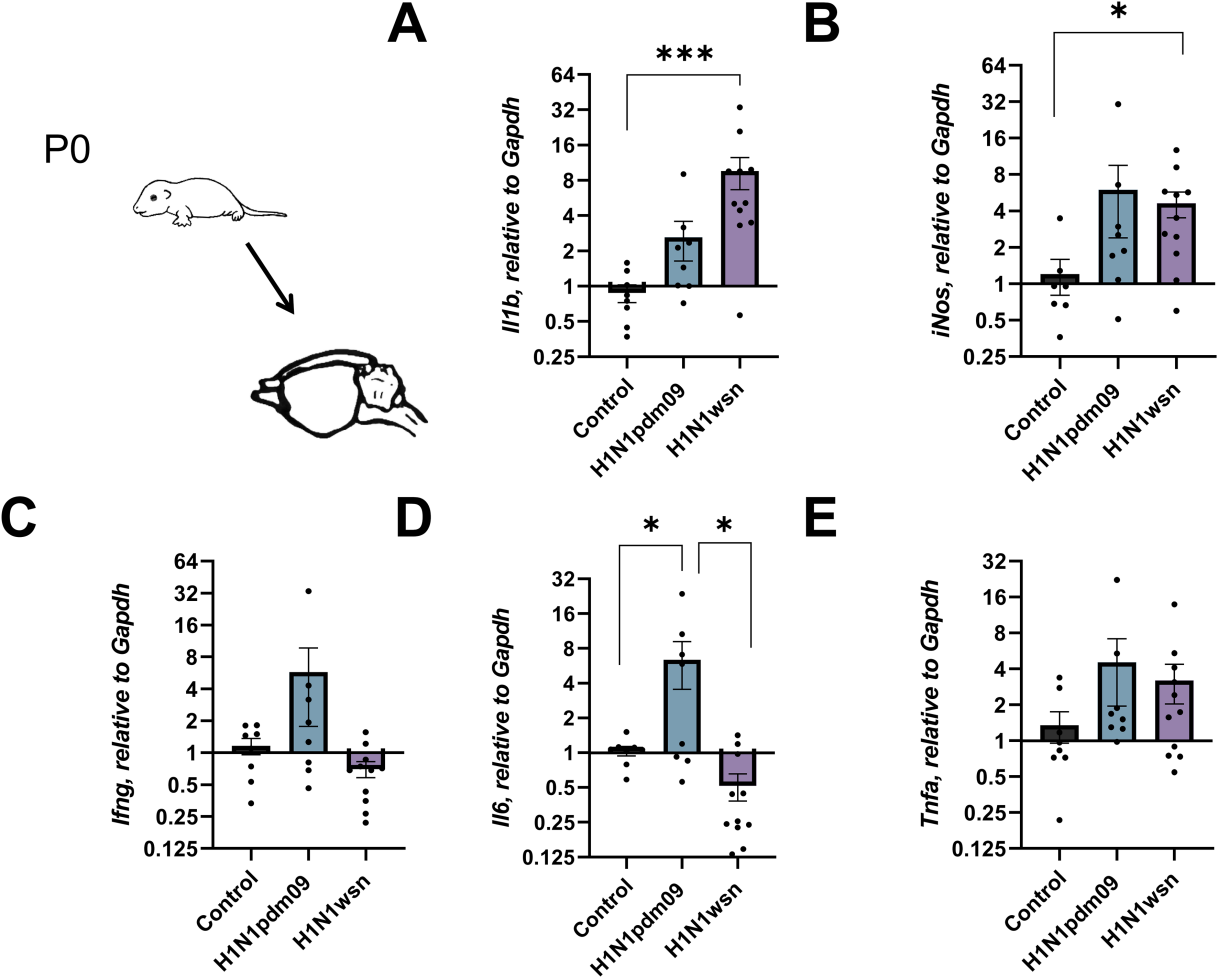
Analysis of mRNA expression of **(A)***Il1b*, **(B)***iNos*, **(C)***Ifng*, **(D)***Tnfa*, **(E)***Il6* in brain tissues of newborn pups (p0) whose mothers were infected with H1N1pdm09 or H1N1wsn. The offspring of influenza infected pregnant mice was used for the genes expression assessment; 4 independent litters; H1N1pdm09–10 pups, H1N1wsn – 11. Mock infected group consists of 3 independent litters, 8 pups. Data are presented as mean ± SD. *p < 0.05; **p<0.01; ***p<0.001. Statistical analysis was performed using one-way ANOVA with Tukey’s test for multiple comparisons.

The mRNA level of cytokines, inflammatory and neurotropic factors in the brain and hippocampal tissues of newborn pups (p0) were evaluated (**Figures 2**, **3** respectively). There was a distinct increase in the expression of the *Il1b*, *iNos*, and *Ifng* in the hippocampus in the case of H1N1wsn infection (**Figures 2A–D**). The pups whose mothers had been infected with H1N1pdm09 showed a significant increase in *Il6* expression (**Figure 2D**). There was also a tendency to increase the level of *Tnfa* mRNA expression in pups hippocampal (**Figure 2E**) and brain (**Figure 3E**) tissues.

The expression of *Il1b* and *iNos* was also elevated in the total brain (**Figures 3A** and 3B respectively), but there was no tendency to increase *Ifng* expression (**Figure 3C**). As in hippocampi, mother’s infection with H1N1pdm09 was associated with *Il6* upregulation (**Figure 3D**) in total brain tissues.

Evaluation of the expression of neurotropic factors *Bdnf* and *Sox2* revealed no significant differences in hippocampi (**Supplementary Figure S4**).

### 2.3 Neurogenesis decreases in the hippocampi of pups (p0) induced by H1N1 infection

**Figure 4:**
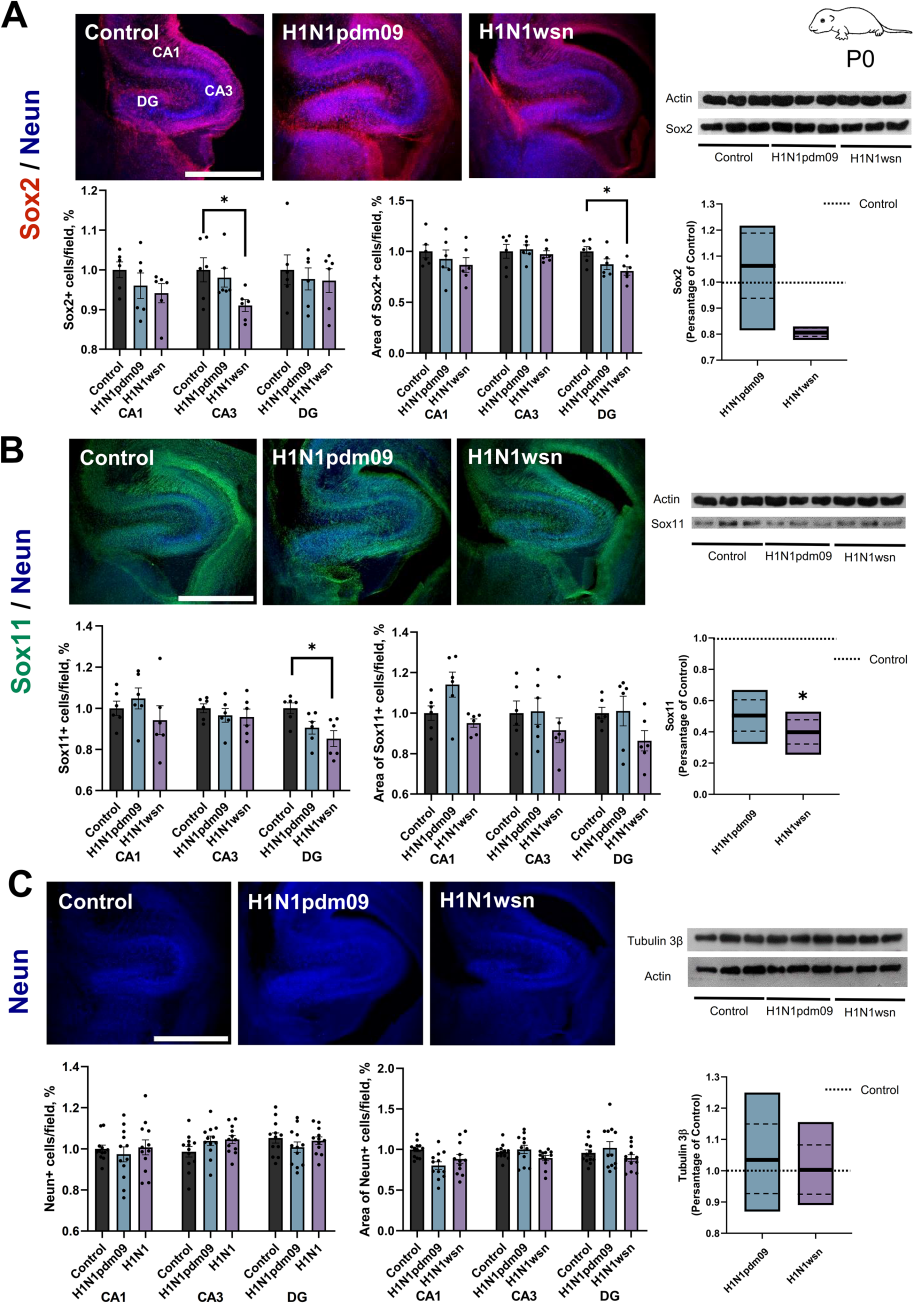
Neurogenesis decreases in the hippocampi of pups (p0) induced by H1N1 infection. Representative images Sox2 (magenta) **(A)**, Sox11 (green) **(B)** and Neun (blue) **(C)** in the frontal slice of the hippocampus mice (P0) whose mothers were infected with the H1N1pdm09 or H1N1wsn influenza strain. Scale bar: 500 um. Quantitation of the number of Sox2-positive (Sox2+) **(A)** Sox11-positive (Sox11+) **(B)** Neun-positive cells (Neun+) **(C)** and their respective areas in the hippocampal regions CA1, CA3, and the dentate gyrus (DG) (N = 3, n=6, Tukey’s test). The changes in Sox2 **(A)**, Sox11 **(B)** and Tubulinβ3 **(C)** expression among the different groups were confirmed with Western blot experiments (n = 6 hippocampus per group, Tukey’s test). The full-length blot is shown, and quantification of protein expression was normalized with actin loading controls and expressed as a percentage of Control groups. Data are presented as mean ± SEM. *p < 0.05.

Influenza virus infection, particularly with highly pathogenic strains such as H1N1, can induce inflammatory processes in the central nervous system (Bauer et al., 2023). However, the impact of such infections on the offspring’s nervous system, particularly in neurogenesis, remains poorly understood. Virus-induced inflammation is hypothesized to disrupt neurogenesis in vulnerable regions such as the hippocampus. The transcription factor Sox2 was selected as a key marker to analyze neurogenesis across different stages due to its critical role in maintaining neural stem cells and progenitors (Zhang and Cui, 2014). Data analysis revealed a reduction in the number of Sox2-positive (Sox2+) cells in the CA3 subregion of the hippocampus (**Table 1**) and a decrease in Sox2+ cells area within the DG subregion of the hippocampus in the H1N1wsn-infected group (**Figure 4A**). For further investigation of neurogenesis, the transcription factor Sox11 was analyzed. Sox11 is involved in the early stages of neurogenesis, including the differentiation of neural stem cells into neurons (Wang et al., 2013). Quantification of Sox11+ cells showed a reduction in the DG region of the hippocampus in pups born from H1N1wsn-infected mothers (**Figure 4B**). Western blot analysis of hippocampus tissues from different groups of mice further confirmed changes in Sox11 protein expression. NeuN was used as a marker to assess mature neurons (Duan et al., 2016). NeuN analysis did not reveal significant differences in the hippocampus’s CA1, CA3, and DG regions between the studied groups based on the selected parameters (**Figure 4C**). Western blot analysis of hippocampus tissues from different groups of mice further confirmed no changes in Tubulinβ3 protein expression, consistent with the immunofluorescence data. Thus, these findings suggest that maternal H1N1 infection disrupts early and intermediate stages of neurogenesis in the hippocampus of offspring, potentially affecting brain development and function.

**Table 1:** Results of the evaluation of neurodevelopment in offspring (P0) whose mothers were infected with H1N1pdm09 or H1N1 influenza.

P0		IHC						WB
Criterion		CA1		CA3		DG		
		Number of cells/field, %	Area of cells/field, %	Number of cells/field, %	Area of cells/field, %	Number of cells/field, %	Area of cells/field, %	Expression/actin
Sox2	Control	0.821 ± 0.016	0.145 ± 0.009	0.797 ± 0.024	0.141 ± 0.009	0.818 ± 0.031	0.187 ± 0.008	1.018 ± 0.089
	H1N1pdm09	0.789 ± 0.026	0.134 ± 0.013	0.781 ± 0.019	0.144 ± 0.006	0.799 ± 0.022	0.163 ± 0.009	1.060 ± 0.116
	H1N1wsn	0.773 ± 0.019	0.126 ± 0.011	0.725 ± 0.012*	0.137 ± 0.004	0.796 ± 0.025	0.151 ± 0.007*	0.832 ± 0.162
Sox11	Control	0.744 ± 0.026	0.145 ± 0.005	0.750 ± 0.016	0.163 ± 0.009	0.880 ± 0.023	0.174 ± 0.005	0.642 ± 0.142
	H1N1pdm09	0.779 ± 0.038	0.165 ± 0.009	0.724 ± 0.025	0.164 ± 0.0105	0.797 ± 0.027	0.176 ± 0.0128	0.542 ± 0.122
	H1N1wsn	0.700 ± 0.052	0.137 ± 0.003	0.717 ± 0.029	0.149 ± 0.103	0.0750 ± 0.033*	0.150 ± 0.009	0.711 ± 0.125*
Neun/Tubulin β3	Control	0.711 ± 0.012	0.157 ± 0.005	0.691 ± 0.018	0.147 ± 0.004	0.791 ± 0.014	0.159 ± 0.008	1.063 ± 0.187
	H1N1pdm09	0.692 ± 0.026	0.126 ± 0.007	0.728 ± 0.016	0.153 ± 0.008	0.758 ± 0.019	0.169 ± 0.013	1.149 ± 0.124
	H1N1wsn	0.716 ± 0.026	0.139 ± 0.031	0.733 ± 0.012	0.136 ± 0.005	0.782 ± 0.016	0.147 ± 0.006	1.111 ± 0.091
GAFP	Control	0.398 ± 0.027	0.039 ± 0.002	0.349 ± 0.032	0.048 ± 0.005	0.35 ± 0.03	0.055 ± 0.005	0.478 ± 0.085
	H1N1pdm09	0.329 ± 0.015	0.048 ± 0.001*	0.291 ± 0.029	0.044 ± 0.005	0.305 ± 0.012	0.044 ± 0.007	0.870 ± 0.105*
	H1N1	0.327 ± 0.045	0.042 ± 0.003	0.33 ± 0.06	0.048 ± 0.002	0.324 ± 0.044	0.049 ± 0.004	1.077 ± 0.176*
Iba1	Control	0.180 ± 0.025	0.028 ± 0.003	0.180 ± 0.013	0.033 ± 0.001	0.160 ± 0.030	0.034 ± 0.005	0.828 ± 0.198
	H1N1pdm09	0.100 ± 0.018	0.019 ± 0.002	0.088 ± 0.011	0.021 ± 0.002	0.120 ± 0.018	0.026 ± 0.003	0.968 ± 0.069
	H1N1wsn	0.100 ± 0.012	0.017 ± 0.002	0.083 ± 0.012	0.015 ± 0.001	0.096 ± 0.012	0.022 ± 0.002	0.636 ± 0.239

Data are expressed as the mean ± SEM. Statistical significance was determined by using a Tukey’s test (*p < 0.05 compared to control).

### 2.4 Neurogenesis decreases in the hippocampi of 14-day-old pups triggered by H1N1pdm09 infection

**Figure 5:**
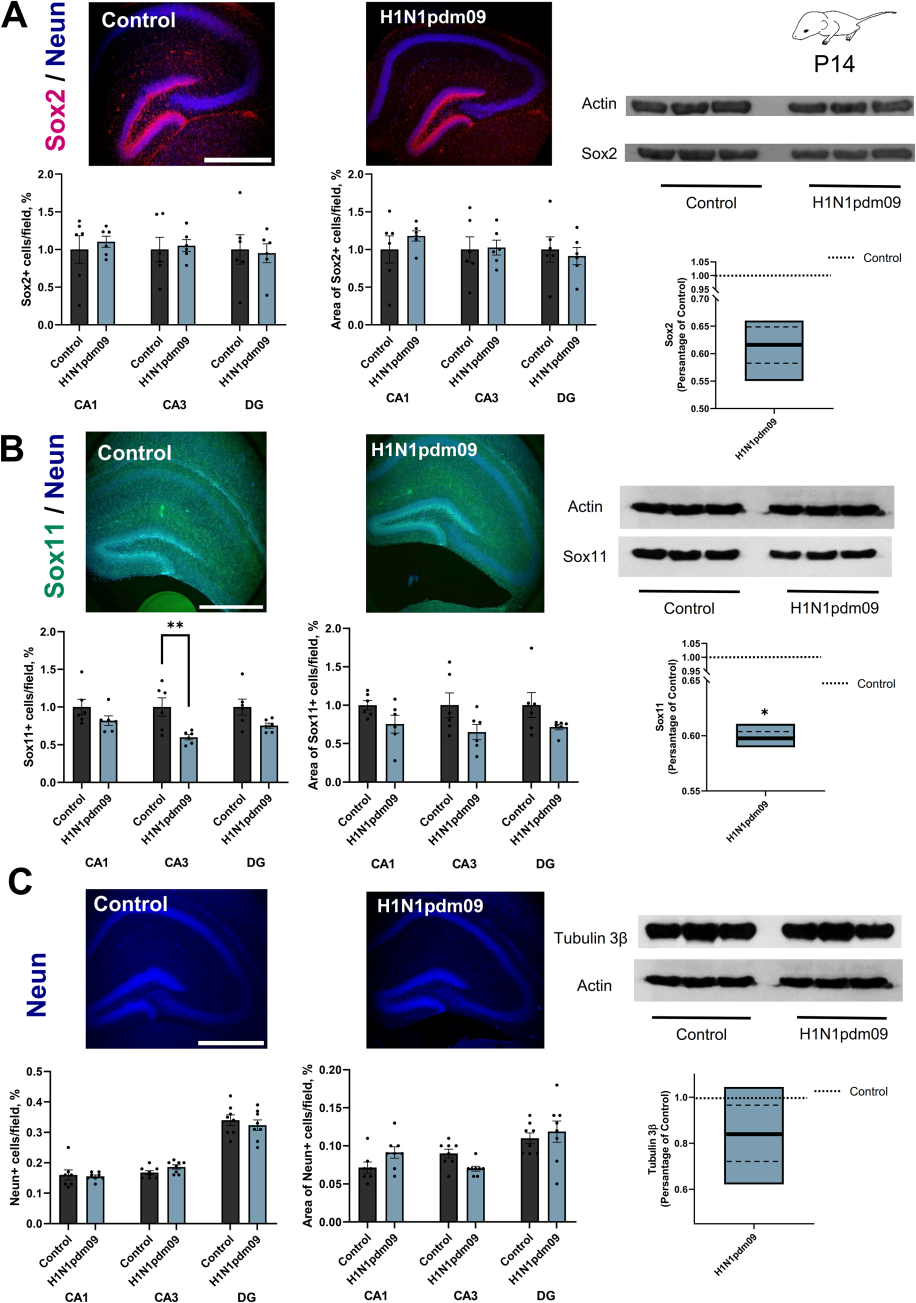
Neurogenesis decreases in the hippocampus of pups (P14) triggered by H1N1pdm09 infection. Representative images Sox2 (magenta) **(A)**, Sox11 (green) **(B)** and Neun (blue) **(C)** in the frontal slice of the hippocampus mice (P14) whose mothers were infected with the H1N1pdm09 influenza strain. Scale bar: 500 um. Right panel: the number of Sox2-positive (Sox2+) **(A)** Sox11-positive (Sox11+) **(B)** Neun-positive (Neun+) cells **(C)** and their respective areas in the hippocampal regions CA1, CA3, and the dentate gyrus (DG) (N = 3, n=6, t-test). The changes in Sox2 **(A)**, Sox11 **(B)** and Tubulin3β **(C)** expression among the different groups were confirmed with Western blot experiments (n = 6 hippocampus per group, t-test). The full-length blot is shown, and quantification of protein expression was normalized with actin loading controls and expressed as a percentage of Control groups. Data are presented as mean ± SEM. *p < 0.05, **p < 0.01.

Due to the high mortality observed among neonatal mice whose mothers were infected with the highly pathogenic H1N1wsn influenza strain, subsequent analyses on the 14th day of life were limited to the mice whose mothers were infected with the H1N1pdm09 influenza strain (**Figure 1**). An analysis of the data revealed that the number of Sox2-positive (Sox2+) cells and their respective areas in the hippocampal regions CA1, CA3, and the dentate gyrus (DG) showed no statistically significant differences between the experimental groups (**Figure 5A**). Western blot analysis of hippocampus tissues from different groups of mice further confirmed no changes in Sox2 protein expression (**Table 2**). However, quantification of Sox11-positive (Sox11+) cells, along with the area occupied by these cells (**Figure 5B**), indicated a reduction in Sox11 protein expression in the CA3 region of 14-days-old mice whose mothers were infected with the H1N1pdm09 influenza virus compared to the control group. Western blot analysis of hippocampus tissues from different groups of mice further confirmed changes in Sox11 protein expression. Sox11 is a critical transcription factor involved in neurogenesis and neuronal differentiation, and its reduced expression may reflect alterations in developmental processes specific to the CA3 hippocampal subregion. Additionally, the expression of NeuN, a marker for mature neurons, was evaluated in the same hippocampal subregions (CA1, CA3, and DG) (**Figure 5C**). The analysis revealed no statistically significant differences in NeuN expression between the experimental groups, suggesting maternal H1N1pdm09 infection did not markedly affect the overall maturation and density of neurons in these regions. Western blot analysis of hippocampus tissues from different groups of mice further confirmed no changes in Tubulinβ3 protein expression, consistent with the immunofluorescence data.

**Table 2:** Results of the evaluation of neurodevelopment in offspring (P14) whose mothers were infected with H1N1pdm09 or H1N1 influenza.

P14		IHC						WB
Criterion		CA1		CA3		DG		
		Number of cells/field, %	Area of cells/field, %	Number of cells/field, %	expression/actin	Number of cells/field, %	Area of cells/field, %	Expression/actin
Sox2	Control	0.180 ± 0.036	0.031 ± 0.005	0.190 ± 0.031	0.033 ± 0.006	0.20 ± 0.04	0.040 ± 0,007	1.051 ± 0.185
	H1N1pdm09	0.190 ± 0.013	0.034 ± 0.003	0.200 ± 0.015	0.035 ± 0.003	0.190 ± 0.026	0.035 ± 0.005	0.648 ± 0.035
Sox11	Control	0.19 ± 0.02	0.029 ± 0.002	0.200 ± 0.024	0.029 ± 0.005	0.340 ± 0.036	0.050 ± 0.008	1.017 ± 0.124
	H1N1pdm09	0.160 ± 0.012	0.022 ± 0.003	0.120 ± 0.006**	0.019 ± 0.003*	0.250 ± 0.011	0.038 ± 0.002	0.608 ± 0.007*
Neun/Tubulin β3	Control	0.160 ± 0.017	0.071 ± 0.007	0.168 ± 0.006	0.090 ± 0.006	0.340 ± 0.017	0.110 ± 0.007	1.046 ± 0.094
	H1N1pdm09	0.156 ± 0.005	0.091 ± 0.007	0.186 ± 0.007	0.070 ± 0.003	0.324 ± 0.017	0.119 ± 0.014	0.881 ± 0.128
GFAP	Control	0.160 ± 0.008	0.030 ± 0.003	0.180 ± 0.017	0.032 ± 0.003	0.180 ± 0.025	0.034 ± 0.002	0.575 ± 0.099
	H1N1pdm09	0.240 ± 0.027*	0.047 ± 0.005*	0.180 ± 0.014	0.034 ± 0.004	0.270 ± 0.034*	0.050 ± 0.004**	0.925 ± 0.056*
Iba1	Control	0.160 ± 0.035	0.023 ± 0.003	0.140 ± 0.025	0.020 ± 0.002	0.200 ± 0.042	0.029 ± 0.004	0.930 ± 0.169
	H1N1pdm09	0.190 ± 0.041	0.026 ± 0.003	0.190 ± 0.038	0.027 ± 0.003	0.350 ± 0.096	0.039 ± 0.005	0.719 ± 0.027

Data are expressed as the mean ± SEM. Statistical significance was determined by using a t-test (*p < 0.05, **p < 0.001, compared to control).

### 2.5 Astrocyte reactivity increases in the hippocampus of neonatal pups and 14-day-old pups induced by H1N1pdm09 infection

**Figure 6:**
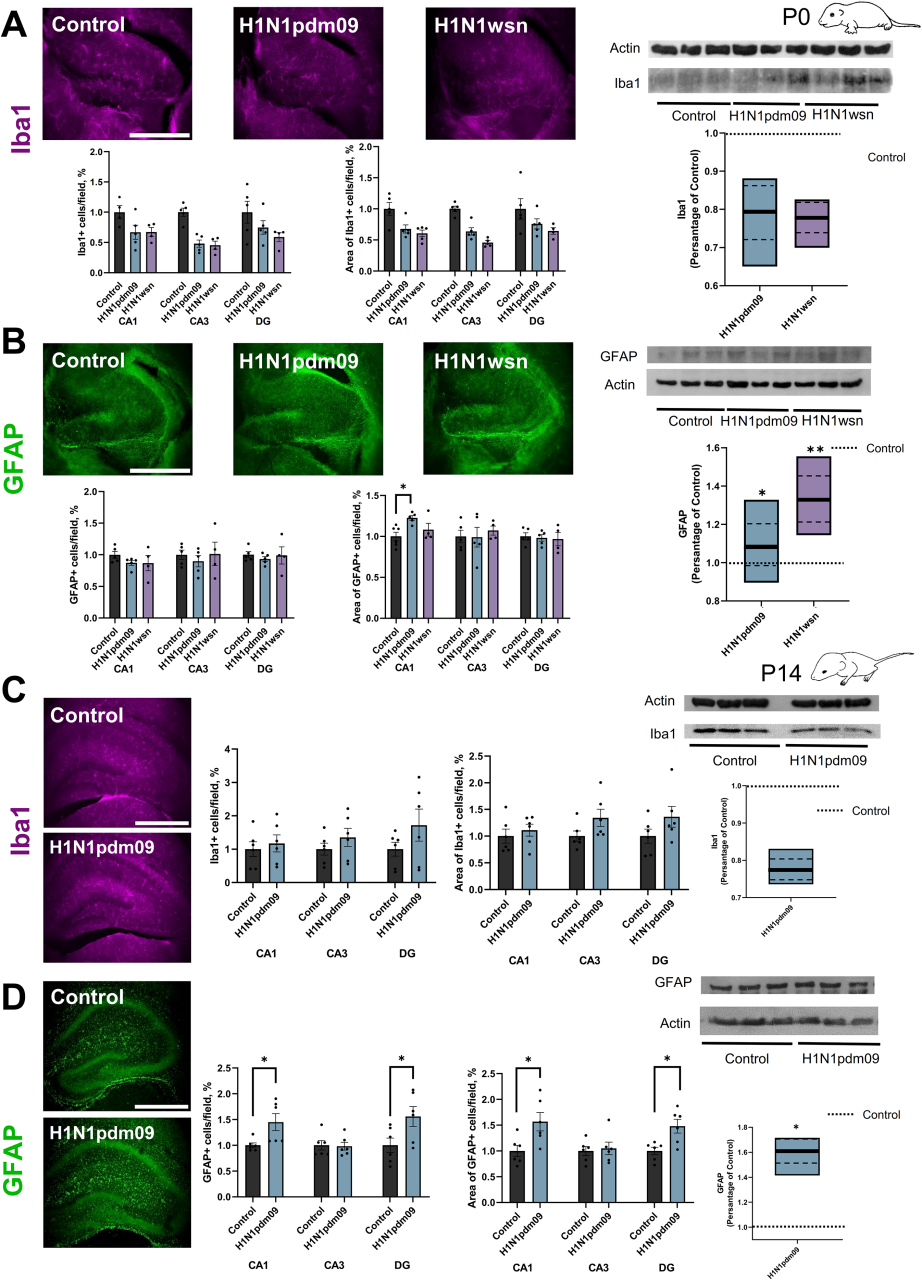
Astrocyte reactivity increases in the hippocampus of neonatal pups and 14-day-old pups induced by H1N1pdm09 infection. Left panel, Representative images Iba1(purple) **(A)** and GFAP (green) **(B)** in the frontal slice of the hippocampus mice (P0) whose mothers were infected with the H1N1pdm09 or H1N1wsn influenza strain, Iba1(purple) **(C)** and GFAP (green) **(D)** in the frontal slice of the hippocampus mice (P14) whose mothers were infected with the H1N1pdm09 influenza strain. Scale bar: 500 um. Right panel: the number of Iba1-positive (Iba1+) **(A, C)** GFAP-positive (GFAP+) **(B, D)** cells and their respective areas in the hippocampal regions CA1, CA3, and the dentate gyrus (DG) (N = 3, n=6, Tukey’s test and t-test). The changes in Iba1 **(A, C)**, GFAP **(B, D)** expression among the different groups were confirmed with Western blot experiments (n = 6 hippocampus per group, Tukey’s test and t-test). The full-length blot is shown, and quantification of protein expression was normalized with actin loading controls and expressed as a percentage of Control groups. Data are presented as mean ± SEM. *p < 0.05, **p < 0.01.

Inflammation is triggered by pathogens such as viruses, initiating various biochemical cascades aimed at restoring homeostasis and eliminating threats. However, in the brain, neuroinflammation is characterized by elevated levels of pro-inflammatory cytokines, increased activation and numbers of microglia, and infiltration of peripheral leukocytes. These processes can lead to neuronal damage (Woodburn et al., 2021). Microglia, the brain’s resident immune cells, are key players in neuroinflammation, and their activation can be visualized using the marker protein Iba1, which is expressed upon microglial activation. In this study, we investigated microglial responses in neonatal mice (P0) whose mothers were infected with the H1N1pdm09 influenza virus. Quantitative analysis of microglia in hippocampal subregions CA1, CA3, and the dentate gyrus (DG) showed no significant changes in the number or area occupied by Iba1-positive (Iba1+) microglia in these regions (**Figures 6A, C**). An analysis of the data revealed that the number of Iba1-positive (Iba1+) microglia cells and their respective areas in the hippocampal regions CA1, CA3 and DG of neonatal mice (P0) showed no statistically significant differences between the experimental groups (**Figure 6A**). Western blot analysis of hippocampus tissues from different groups of mice further confirmed no changes in Iba1 protein expression. Owing to the high mortality of neonatal mice born to mothers infected with the highly pathogenic H1N1wsn influenza strain, analyses at postnatal day 14 were conducted only in mice whose mothers had been infected with the H1N1pdm09 strain (**Figure 1**). Also, there were no changes in the group of 14-day-old mice whose mothers were infected with H1N1pdm09 compared to the control group, as confirmed by western blot analysis (**Figure 6C**).

In addition to microglia, astrocytes are crucial regulators of innate and adaptive immune responses in the central nervous system (CNS). Depending on the context and timing, astrocyte activity can exacerbate inflammatory responses and tissue damage or contribute to immunosuppression and tissue repair (Colombo and Farina, 2016). Reactive astrocytes are often characterized by a dramatic increase in GFAP immunoreactivity, a phenomenon known as astrogliosis (Wilhelmsson et al., 2006). To evaluate astrocyte reactivity, brain tissues from all experimental groups were stained for GFAP, a marker of astrocytic activation. The fluorescence intensity of GFAP staining was analyzed as an indicator of astrocyte reactivity in the hippocampal subregions CA1, CA3, and DG. In the CA1, the area occupied by GFAP-positive (GFAP+) astrocytes increased in the group of neonatal mice (P0) whose mothers were infected with H1N1pdm09 compared to the control group (**Figure 6B**). Western blot analysis of hippocampus tissues from different groups of mice further confirmed changes in GFAP protein expression, consistent with the immunofluorescence data. According to the Western blot results, GFAP protein expression was also increased in the group of neonatal mice (P0) whose mothers were infected with H1N1wsn compared to the control group. Quantification of the number and area of GFAP+ astrocytes in the CA1 and DG regions of the hippocampus revealed that both parameters were significantly increased in 14-day-old mice whose mothers were infected with H1N1pdm09 compared to control (**Figure 6D**). Western blot analysis of hippocampus tissues from different groups of mice further confirmed changes in GFAP protein expression, consistent with the immunofluorescence data. These findings suggest that maternal H1N1pdm09 influenza infection induces localized astrocytic reactivity in specific hippocampal subregions of neonatal mice.

## 3 Discussion

Due to the similar hemochorial structure of human and mouse placentas, murine models of influenza infection are a common experimental approach to reproduce influenza infection in pregnant women. In mouse models, infection with IAV leads to a decrease in maternal weight gain during pregnancy, a shortening of pregnancy and premature birth as well as a decrease in the number of newborn pups and their body weight (Pazos et al., 2012; Littauer et al., 2017), and an increase in the incidence of stillbirths from 2% to 50% (Littauer et al., 2017). Influenza infection also causes damage of pulmonary epithelium, production of reactive oxygen species, activation of cyclooxygenase-2 and increased production of prostaglandin E2 (Littauer et al., 2017). In this study we use two influenza A strains (subtype H1N1) that are often utilized in virological experiments. For instance, mouse adapted strain A/California/07/2009 (H1N1pdm09) was shown to induce maternal mortality and morbidity, severe pneumonia with immune cells infiltration; miscarriage, as well as reduced serum and uterine progesterone; elevated lungs IL-1α, IL-6, G-CSF, and chemokines in pregnant BALB/c mice (Kim et al., 2012). In our work it was shown that influenza infection resulted in significant mortality in newborn pups; H1N1wsn induced the death of an overwhelming number of pups within two weeks after birth. However, the body weight dynamics did not show distinct developmental delay in surviving pups.

Infection with IAV leads to a distinct immune response in the lungs, production of pro-inflammatory cytokines and chemokines (IL-1b, IL-6, G-CSF, KC, MIP-1b), and decreased bronchodilation (Littauer et al., 2017). The situation is aggravated by the delayed recovery of the pulmonary epithelium after influenza infection (Marcelin et al., 2011). We detected elevated expression of IFN-inducible *Oas-1a/g* and *Irf-7* in mother’s lungs at the mRNA level after childbirth, corresponding to 5–7 days after influenza infection, and indicating the innate immune response activation in response to viral infection.

The development of neurodegenerative diseases in offspring can be associated with the activation of cytokines and inflammatory factors. Influenza-induced immune activation in pregnant mothers is not always associated with an increased risk of developmental delays in offspring if the mother never had a fever at the time of infection (Lee et al., 2019). Therefore, fever-associated acute phase cytokines IL-1ß, IL-6, IL17, TNF-α are considered to play the major role in the development of neuropathological processes (Elgueta et al., 2022). Using RT-PCR, we measured the mRNA levels of the *Il1b*, *iNos*, *Il6, Ifng*, and *Tnfa* in the hippocampal and brain tissues of newborn pups. We noted the increased expression of *Il1b*, *iNos*, *Ifng* in the case of H1N1wsn, and *Il6* in the case of H1N1pdm09 infection. This way, influenza-induced maternal immunity could stimulate cytokines and proinflammatory factor expression in the brains of newborn pups.

Elevated levels of IL-1ß may be linked to the development of hypoxia, activation of the immune system (Lee et al., 2019), and inflammatory processes in fetal brain (Elgueta et al., 2022). Injection of IL-1ß in sub-chronic doses resulted in increased mortality, placental and fetal brain cortical thinning, and neurodevelopmental deficits (Lee et al., 2019). Intrauterine injected IL-1ß stimulates the production of pro-inflammatory cytokines in fetal brain (IL-1β, TNFα, CXCL-5, CXCL-10, and CXCL-11), it also induces cytokines, hypoxia-associated factors (iNOS and HIF1α), as well as oxidative stress proteins in the placenta (Lee et al., 2019). Intrauterine injections with lipopolysaccharide on day 15 of gestation resulted in cortical neuronal injury in pups. The neurotoxicity was associated with increased NMDAR1, but not nNOS (neuronal nitric oxide synthase) mRNA level. Prenatal administration of IL-1 receptor antagonist downregulated inflammation-induced NMDAR1 expression, blocked nNOS activation in brain cortex and prevented delayed cortical neurotoxicity (Leitner et al., 2014). IL-1ß was shown to enhance iNOS expression in brain tissues (Licinio et al., 1999) that can be related with elevated NO level and neurodegeneration (Papageorgiou et al., 2016). This way, we suggest that maternal influenza infection could result in increased IL-1ß and iNOS expression and disruption of neurodevelopmental processes.

Maternal influenza infection can result in inflammation in the fetal tissues and a lack of nutrients in the developing fetus (Littauer et al., 2017). Therefore, we measured pro-inflammatory factors expression in the liver. We noted that only IL-1β expression was slightly increased. Based on our results, it is difficult to conclude whether fetal brain inflammation is the result of maternal cytokines penetration through the placenta, trophoblast activation, or the reaction of fetal immunity. However, the absence of excessive IL-6, TNFα, and iNOS expression in the liver point to limited inflammation in the periphery. Several strains of influenza A virus are known to infect specific subpopulations of neurons in the mouse brain. Influenza strain A/WSN/33 is considered to be neurotropic since viral vRNA and mRNA persist in olfactory bulbs of immunodefective mice (Aronsson et al., 2001).

Neurovirulent strains can cross the CNS through the olfactory, vagus, trigeminal, and sympathetic nerves, whereas non-neurotropic strains are only able to activate the immune response, having an impact on neurotrophic (BDNF, NGF) and immunomodulatory (CD200, CXCL1) factors within the hippocampus and increasing the microglial reactivity (Jurgens et al., 2012). Therefore, neurotropic and non-neurotropic IAV strains might utilize various mechanisms to damage growing fetal brain. We used two different neurovirulent and non-neurovirulent strains in the study.

**Figure 7:**
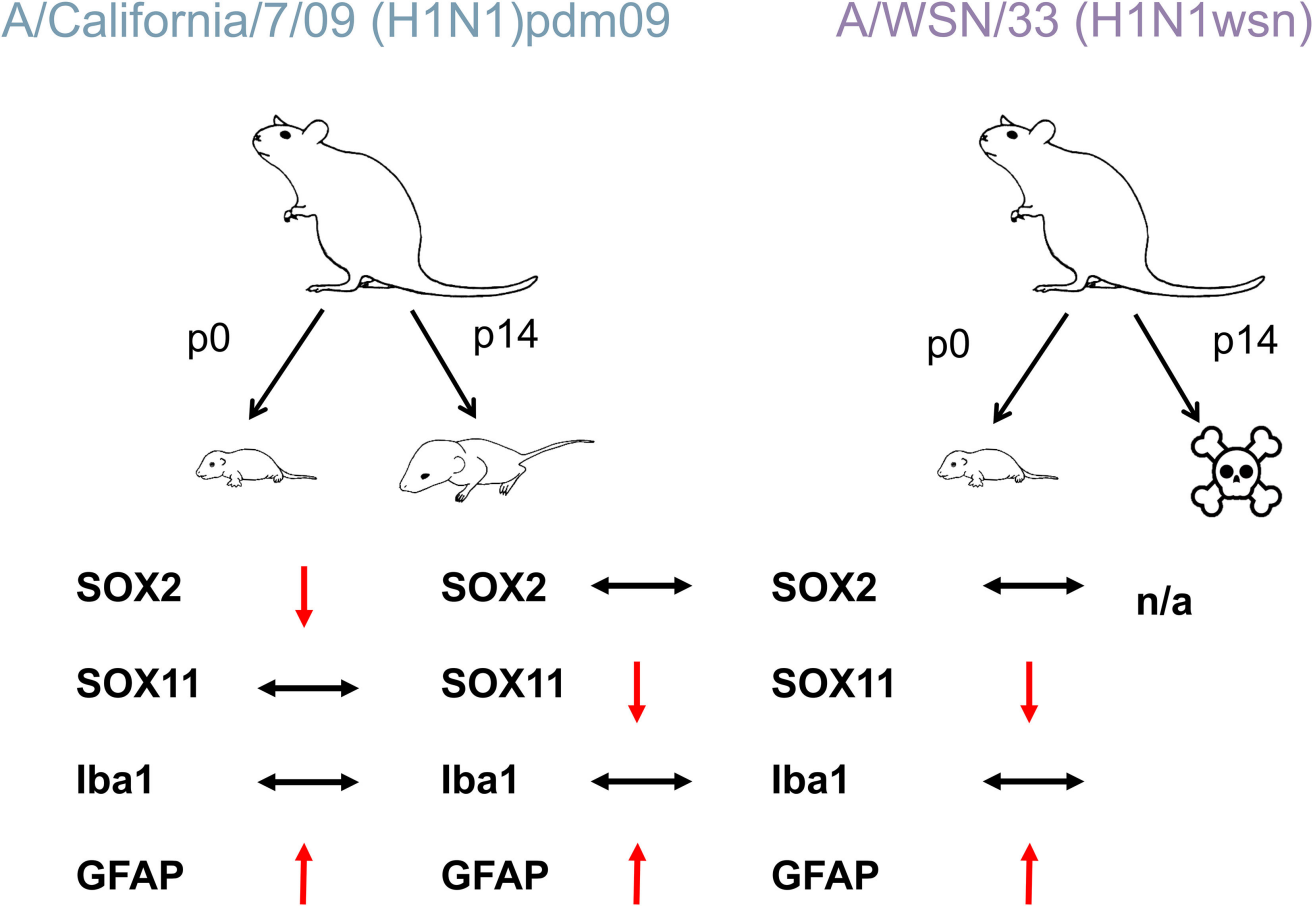
Model of the neurotropic effects in the offspring of mothers infected with H1N1pdm09 and H1N1wsn influenza virus. The left panel represents infection with H1N1pdm09, while the right panel shows infection with H1N1wsn. Arrows indicate the progression from p0 to p14, with a skull and crossbones symbol marking lethality at p14 for H1N1wsn. Several cellular markers were analyzed: SOX2 (neural progenitor marker) remained unchanged (↔), SOX11 (neuronal differentiation marker) was downregulated (↓) at p14 in both virus strains, Iba1 (microglia marker) showed no significant change (↔), and GFAP (astrocyte marker) was upregulated (↑) at both time points. The results indicate that H1N1pdm09 alters neuronal and glial marker expression without affecting survival, while H1N1 leads to severe effects, including lethality at p14, with similar trends in marker changes before death. This figure highlights the differential neurotropic effects of H1N1 virus strains in developing mice, with significant astrocyte activation (GFAP ↑) and decreased neuronal differentiation (SOX11 ↓).

Our study reveals significant disruptions in hippocampal neurogenesis and increased astrocytic reactivity in the offspring of mothers infected with H1N1wsn and H1N1pdm09 influenza viruses. These findings suggest maternal influenza infection induces long-term alterations in the developing brain, with potential implications for neurodevelopmental outcomes. The observed reductions in Sox2+ and Sox11+ cells highlight a marked impact of maternal infection on early and intermediate stages of neurogenesis in the hippocampus (**Figures 5**, **7**; **Table 2**). Sox2, a transcription factor critical for maintaining neural stem cells (Pevny and Nicolis, 2010), exhibited reduced expression in neonatal pups’ (P0) CA3 and DG regions, indicating compromised neural progenitor populations. This aligns with previous findings that maternal inflammation can disrupt hippocampal development during critical periods (Knuesel et al., 2014; Zhang and Cui, 2014; Hagberg et al., 2015). The vulnerability of Sox2 expression to maternal immune activation may be mediated by inflammatory cytokines released in response to infection. Elevated maternal inflammation has been shown to cross the placenta and impact fetal brain development, altering key signaling pathways involved in stem cell maintenance and proliferation (Bauer et al., 2023). This aligns with our findings, as the hippocampal subregions most affected—CA3 and DG—are highly susceptible to inflammation-induced damage due to their reliance on active neurogenesis during development. Interestingly, despite the reduction in Sox2+ cells, NeuN expression, a marker of mature neurons, remained unaffected in both neonatal (P0) and juvenile (P14) mice. This suggests that while the progenitor pool is disrupted, there may not yet be a sufficient population of mature neurons at these early developmental stages to observe significant effects. Alternatively, compensatory mechanisms, such as increased proliferation or differentiation of remaining progenitors, may mitigate the impact on mature neurons in the short term.

Reduced Sox11+ cells in the dentate gyrus (DG) and CA3 regions in P0 and P14 pups suggest impaired differentiation of neural stem cells into neurons. Sox11 is a critical transcription factor involved in the transition of neural progenitors to neuronal lineages (Stevanovic et al., 2021). Interestingly, beyond its role in neurogenesis, Sox11 also plays a pivotal role in promoting endothelial cell inflammation, where it modulates pro-inflammatory signaling pathways and cytokine expression (Ni et al., 2025). This dual role suggests that Sox11 could serve as a key mediator linking systemic inflammation to disruptions in neurogenesis. Elevated inflammation during pregnancy may reduce Sox11 expression in neural progenitors while simultaneously contributing to vascular and endothelial changes in the developing brain, further exacerbating neurodevelopmental deficits. It should be noted that although Sox11 may contribute to vascular changes, this was not assessed in the present study. The shared mechanisms of SOX11 in inflammation, tissue remodeling, and disease progression suggest it is a potential therapeutic target in inflammatory and cancer-related pathologies (Tsang et al., 2020).

This study includes several limitations related to the mouse cohorts that warrant consideration. First, our analyses were conducted using mixed-sex groups, which may limit the ability to generalize the findings to males and females individually. Because sex differences in brain aging and longevity are well established, future studies should incorporate enough of both sexes to better capture these biological distinctions. Second, some experimental groups included relatively few animals. Variation in group size and the number of biological replicates arose from the need to distribute pups from each litter across three parallel assays (IHC, Western blot, and PCR). This design ensured that all pups originated from the same virus-exposed mother but inherently constrained the number available for each experiment. Finally, a time-course analysis would further clarify the progression and temporal dynamics of neurogenesis decline across different developmental windows. Follow-up experiments incorporating additional neurogenesis markers such as BrdU, Ki67, and COX-2 would help pinpoint the specific stage at which the neurogenic lineage is altered in genetically diverse mice. Although our study did not concentrate on cell proliferation, these markers would clarify whether changes arise from differences in the size of the stem cell pool, delays in stem-cell activation, or increased cell loss during maturation.

Neuroinflammation plays a central role in the disruption of neural development, and both astrocytes and microglia are key mediators of this process. In our analysis, we did not observe significant changes in the number or area occupied by Iba1-positive (Iba1+) microglia in the hippocampal subregions CA1, CA3, and the dentate gyrus (DG) in neonatal mice (P0) whose mothers were infected with the H1N1pdm09 virus. The lack of significant microglial activation in P0 pups indicates that the immediate postnatal period may not be as sensitive to the effects of maternal infection compared to later developmental stages. However, we acknowledge that microglia are highly complex cells, and our current approach—limited to the assessment of Iba1+ cell density—captures only a portion of their functional diversity. More detailed analyses, such as morphological assessment, co-staining with markers of phagocytic activity, and co-localization with neuronal or neural stem cell markers, would be valuable in future studies to better understand potential subtler changes in microglial function (Paolicelli et al., 2022). However, astrocytes also play a pivotal role in the brain’s immune response, acting as regulators of both the innate and adaptive immune systems (Giovannoni and Quintana, 2020). Depending on the context, astrocyte activation can have protective effects, contributing to tissue repair and immunosuppression, or it can exacerbate neuroinflammation and tissue damage (Colombo and Farina, 2016; Verkhratsky et al., 2023). In our study, we examined GFAP (glial fibrillary acidic protein) immunoreactivity in the hippocampal regions CA1, CA3, and DG to assess astrocytic activation in response to maternal H1N1pdm09 infection. The observed astrocyte activation in the CA1 region at P0 suggests that this subregion is particularly sensitive to the effects of maternal infection during the immediate postnatal period. The CA1 region’s role in memory consolidation (Remondes and Schuman, 2004) and spatial navigation may underlie its heightened sensitivity to immune challenges (Geiller et al., 2023). The persistent and enhanced astrocyte activation in the CA1 and DG regions at P14 indicates that the effects of maternal infection on astrocytes are not transient but rather persist and intensify over time. The DG’s role in neurogenesis and plasticity, along with the CA1’s involvement in memory processes, underscores the potential long-term consequences of astrocyte activation in these regions.

## 4 Conclusion

We discovered that infection of pregnant mice with highly pathogenic H1N1 or H1N1pdm09 influenza strain results in significant morbidity and mortality in the offspring and neuroinflammation. Our study demonstrates that maternal H1N1 infection induces significant disruptions in hippocampal neurogenesis and increases astrocytic reactivity in the offspring, with subregion-specific effects. These findings underscore the need for further research into preventive and therapeutic strategies to mitigate the long-term consequences of maternal infections on offspring brain development.

Our study focused on the influence of maternal influenza infection on neuroinflammation and hippocampal neurogenesis. However, IAV infection during pregnancy is a quite complex process, causing maternal immunity activation, progesterone downregulation, inflammation in the placenta, immunological and pathological changes in fetal tissues (Chan et al., 2010; Littauer et al., 2017). This way, investigation into the influence of influenza infection on fetal periphery can significantly extend our understanding in future.

A proposed mechanism linking maternal immune activation (MIA) to altered neonatal neurodevelopment involves cytokine-driven oxidative stress and disruption of neural progenitor homeostasis. MIA induces a fetal neuroinflammatory milieu characterized by increased IL-1β and iNOS expression, which enhances the production of reactive oxygen species (ROS) in neonatal brain tissue. Pro-inflammatory cytokines such as IL-1β stimulate microglial and astrocytic activation, driving iNOS-derived nitric oxide (NO) synthesis and subsequent formation of peroxynitrite, a highly reactive nitrogen species implicated in oxidative cellular damage (Monif et al., 2016). Elevated ROS may disrupt neural progenitor cell maintenance by impairing transcriptional programs that depend on stemness- and fate-regulating factors such as Sox2 and Sox11. Excessive ROS can induce DNA damage and activate stress pathways: in neural stem cells (NSCs), oxidative stress causes double-strand breaks, triggers DNA damage response (DDR), and leads to senescence or reduced proliferation (Li et al., 2024). In parallel, oxidative stress may cause epigenetic alterations (DNA methylation changes, histone modifications), which can reduce accessibility of chromatin regions controlling stemness-related genes including Sox2/Sox11 (Huang et al., 2022). Moreover, high ROS may interfere with key developmental signaling pathways such as Wnt/β-catenin signaling, redox-sensitive modulators (e.g. redox sensor Nucleoredoxin, NXN) can dissociate from their inhibitory complexes under oxidative conditions, altering Wnt signaling output, which in turn can influence progenitor fate commitment (Brinkmeier et al., 2025). Disruption of Wnt signaling or other pathways like Notch signaling, also known to regulate progenitor maintenance and differentiation may shift the balance away from self-renewal toward differentiation or apoptosis (Bhavanasi and Klein, 2016). While direct data in neural progenitors is limited, such redox-regulated signaling mechanisms are well described in other stem-cell contexts and in progenitor models (Boopathy et al., 2013). Despite progenitor-level vulnerability, mature or post-mitotic neurons may resist ROS-induced cell death by activating antioxidant defenses: for instance, Nrf2, is a master regulator of cellular antioxidant response, has been shown to support survival and redox-homeostasis in NSCs/NPCs under oxidative stress, suggesting potential compensatory mechanisms that preserve neuronal marker expression even when progenitor pools are compromised (Iqbal and Eftekharpour, 2017). At the same time, reactive glial cells such as astrocytes may contribute to chronic oxidative and inflammatory stress: mitochondrial dysfunction or NADPH-oxidase activity in glia can increase ROS production, sustaining a harmful microenvironment for hippocampal neurogenesis and rendering the neurogenic niche vulnerable (Dos Santos et al., 2021).

Together, these observations support a biologically plausible mechanism by which ROS overproduction, for instance following inflammatory insults such as maternal immune activation could impair neural progenitor maintenance (via DNA damage, epigenetic dysregulation, disrupted signaling) and reduce Sox2/Sox11-dependent progenitor populations, while allowing survival of differentiated neurons via antioxidant responses, and promoting chronic neuroinflammation via glia-derived ROS.

## 5 Methods

### 5.1 Viruses

Mouse-adapted influenza strains A/California/7/09 (H1N1)pdm09 and A/WSN/33 (H1N1) [H1N1wsn] were obtained from the Virus and Cell Culture Collection of the Smorodintsev Research Institute of Influenza in St. Petersburg, Russia. The strains were propagated in 11-day-old embryonated chicken eggs, purified using a sucrose gradient, and stored at -80 °C.

### 5.2 Animals

All animal procedures were conducted in compliance with the regulations for the ethical treatment of animals as outlined by the European Convention for the Protection of Vertebrate Animals Used for Experiments and Other Scientific Purposes (Council of Europe, 1986). The study was approved by the Ethical Committee at the Smorodintsev Research Institute of Influenza, Russia (protocol № 62 dated 30.09.2022).

Two-month-old female C57/BL/6 mice were used for the study. The mice were examined daily for copulation plugs. The presence of the plug was considered the first day of pregnancy. Weight dynamics were also monitored to confirm pregnancy. Infection of the mice was carried out on the 14th day of pregnancy (E14).

### 5.3 Influenza infection

In the first stage, the minimum lethal dose of the virus (MLD_50_) was determined in pregnant mice. MLD_50_ represents the dose that causes the death of 50% of infected animals. The viral titers were calculated using the Reed-Muench method. The MLD_50_ value was found to be 10^-4.25^ TCID_50_/100µL for the strain H1N1pdm09 and 10^-3.5^ TCID_50_/100µL for the strain H1N1wsn.

Pregnant mice were infected intranasally with the mouse-adapted strain H1N1wsn or H1N1pdm09 at the dose of 1 MLD_50_ per mouse. A volume of 30 µL of the viral suspension was administered, with the procedure performed under 4-6% v/v diethyl ether anesthesia. Control mice (mock-infected) were treated with 30 µL of sterile PBS. The control group of mice was treated with placebo (PBS); the administration was also performed under mild ether anesthesia. The mice were inhaled with 4-6% v/v diethyl ether. 33 pregnant mice were infected with A/California/7/09 (H1N1)pdm09, 24 were infected with A/WSN/33 (H1N1wsn). Mock infected group consisted of 15 pregnant mice.

Pups were born 5–7 days after viral infection (E19-E21). We randomly sacrificed 2 or 3 pups from four litters (a mixture of males and females) to assess cytokine, pro-inflammatory and neurotrophic factor expression using RT-qPCR. Pup’s brains and hippocampi were isolated and immediately homogenized using a Tissue Lyser II (Qiagen) in Trizol Reagent (Invitrogen, Austin, TX, USA). The hippocampi were extracted from both hemispheres. Brain atlas and stereoscope were used to confirm region specificity.

The mothers of the pups were sacrificed for evaluation of viral RNA level. The mice were euthanized by CO_2_ inhalation, according to AVMA Guidelines for the Euthanasia of Animals, CO_2_ flow rate was 3 L/min. The lungs were isolated and homogenized with the same method.

About 4 neonates from 4 litters were sacrificed, their hippocampi were isolated and utilized for immunohistochemistry and western blotting.

The remaining mothers and pups were weighed daily, and their mortality and morbidity were monitored. After 2 weeks, grown up pups were sacrificed. A detailed scheme of the experiment is presented in **Figure 1**, **Table 3**.

**Table 3:** The number of pups in the experiment.

Experimental group	Mock infected	(H1N1)pdm09	H1N1wsn
Pregnant Mice	15	33	24
Viable pups at P0	64	104	72
IHC & WB at P0	20	15	18
RT-qPCR at P0	8	10	11
Survival curves and Weight	36	79	43

IHC, immunohistochemistry; WB, western blotting.

### 5.4 RT-qPCR

Total RNA was isolated using Trizol Reagent (Invitrogen, Austin, TX, USA), following the manufacturer’s recommendations. One microgram of total RNA was treated with DNase (Promega, Madison, WI, USA) and then directly reverse-transcribed using oligo-dT16 primers and RNAscribe RT reverse transcriptase (BioLabMix, Novosibirsk, Russia). Complementary DNA synthesis was conducted at 50°C for 50 minutes, the products stored at −20°C until use. qPCR was performed using the 2×BioMaster HS-qPCR reagent (BioLabMix, Novosibirsk, Russia) and primers (**Supplementary Table S1**). The primers were able to detect all transcription variants of the genes. Relative expression values were calculated using the ΔΔCt method, with GAPDH serving as the normalization gene.

CDC Influenza A/B Typing Kit (# FluIVD03-1, Centers for Disease Control and Prevention, USA) was used to detect viral RNA. It was validated that the kit was able to detect mouse-adapted strains of influenza, similar to human influenza strains. The fluorescent signal was absent in mock infected animals.

### 5.5 Immunohistochemistry

Mice pups (postnatal days P0–P14, mixed sex) were euthanized by decapitation with sharp surgical scissors or guillotine. The brains of P0 and P14 pups after decapitation were fixed in 4% paraformaldehyde (PFA) for a month. Then the brains were cut into 90 μm and 60 μm thick slices, respectively, at the same depth, using a vibrotome (5100mz Campden Instruments; Leicestershire, England). The resulting slices were placed in a 24-well tablet in 0.5% PFA overnight. The next day, immunohistochemical staining was performed. First, the slices were washed 2 times for 10 min in PBS, then incubated in 20% HCL solution, then washed in 0.1% Tween-20, and the tablet was on a shaker all the time. Next, the slices were incubated in a blocking solution containing 5% bovine serum albumin (BSA), 0.1% Triton X-100 in PBS, for 1 hour at room temperature (RT). After that, the slices were incubated overnight at 4°C with primary antibodies mouse monoclonal anti-SOX2 (1:500, cell signaling - L1D6A2), rat monoclonal anti-SOX11 (1:500, Millipore - MABE1929-25UG), goat polyclonal anti-NeuN (1:1000, Invitrogen - PA5-143586), rabbit polyclonal anti-Iba-1 (1:400, Servicebio - SB-GB114490), and rabbit polyclonal anti-GFAP (1:1000, BioLegend – 840001) diluted in a solution containing 5% BSA, 0.125% Triton X-100 in PBS. The next day, the slices were washed 3 times in PBS for 10 minutes and then incubated at RT for 1.5 hours with secondary antibodies Alexa Fluor 488-conjugated Goat Anti-Mouse IgG (H+L) (1:500, Servicebio - GB25301), Alexa Fluor 488 Goat Anti-Rat IgG H&L (1:500, abcam - ab150157), Alexa Fluor 647 Donkey Anti-Goat IgG H&L (1:500, abcam - ab150131), Alexa Fluor 594 Goat anti-Rabbit IgG (H+L) (1:1000, Invitrogen - R37117) diluted in a solution containing 5% BSA, 0.125% Triton X-100 in PBS. After the slices were washed 3 times in PBS, then they were placed under cover glasses using an Aqua Poly/Mount mounting medium.

### 5.6 Imaging and quantification of cell density

Brain slices were visualized using a confocal microscope (ThorLabs, USA) with a ×10 objective lens. The 10× objective was chosen to allow imaging of the entire region of interest, which is relatively small in mice of the corresponding age. At this developmental stage, glial cells are sparse and not yet fully formed, so higher magnification was not required for accurate quantification. Image parameters included a resolution of 2048×2048 pixels, 1421 μm/pixel, and a step size along the z-axis of 0.5 μm. Images were analyzed using the ImageJ software, and areas of positive cells were identified using a bandpass filter (with uniform settings for all images), automatic binarization (with a universal threshold for all images), and the “Analyze Particles” function. The number of cells and the area occupied by them, normalized per unit area, were considered in the calculations. All data were normalized within each staining to the control group’s mean inoculated with PBS.

### 5.7 Western blot

The hippocampus was extracted from both hemispheres. Hippocampal lysates were separated using 5% polyacrylamide stacking and 4% resolving gels under denaturing conditions, then transferred to a polyvinylidene fluoride (PVDF) membrane (#IPVH00010, Merck Millipore) in transfer buffer (25 mM Tris, 192 mM glycine, 20% methanol (#67-56-1, Merck Millipore), 0.01% SDS) at 25 V for 30 minutes (BioRad). The membrane was blocked for 1 hour in 5% BSA/TBST (tris-buffered saline with 0.1% Tween 20: 50 mM Tris, 150 mM NaCl, 0.1% Tween 20, pH=7.6). After blocking, the membrane was incubated overnight (16 hours) with primary antibodies to SOX-2 (1:1000, cell signaling - L1D6A2), to SOX-11 (1:1000, Millipore - MABE1929-25UG), to Tubulin beta-3 (1:1500, DSHB, E7-c), to Iba-1 (1:2000, Servicebio - SB-GB114490), to GFAP (1:10000, BioLegend – 840001) and to Actin (1:2000, Millipore - MAB1501). The membrane was then washed and incubated with secondary antibodies conjugated with horseradish peroxidase anti-rabbit antibody (1:2000 dilution, DAKO - P0448), or anti-mouse antibody (1:2000 dilution, DAKO - P0447), or anti-rat antibody (1:700, SinoBiological - SSA005). Next, the membrane was washed several times and incubated in a solution that induces a chemiluminescence reaction. The fluorescent signal was detected using X-ray film (CP-BU NEW, #EWPJH, AGFA) according to the manufacturer’s recommendations. Western blot images were analyzed using ImageJ software. The expression level of Actin was used as a control for protein loading in the sample. All data were normalized within each staining to the control group’s mean inoculated with PBS.

### 5.8 Statistical analysis

Statistical analyses were conducted using GraphPad Prism 9 (GraphPad Software, Version 9.0). To determine the distribution, the Shapiro–Wilk test was calculated. If the obtained results followed a normal distribution, the equality of variances was confirmed by assessing the data using the Bartlett test. Subsequently, the data with a normal distribution was analyzed using the Student’s t-test and a standard one-way analysis of variance (ANOVA), followed by the utilization of the Tukey test for *post-hoc* analysis. Survival curve analysis was performed using the Mantel-Cox test. P-values for significance are provided in the figure legends.

## Data Availability

The raw data supporting the conclusions of this article will be made available by the authors, without undue reservation.
