# Supplementary material for: Neurogenesis decreases in the offspring of mothers infected with influenza A virus
Source: Front Cell Infect Microbiol. 2026 Jan 6;15:1704546. doi: 10.3389/fcimb.2025.1704546 (PMC12816302; doi:10.3389/fcimb.2025.1704546)
Supplement: Supplementary file 1 [file SupplementaryFile1.pdf]

SUPPLEMENTARY

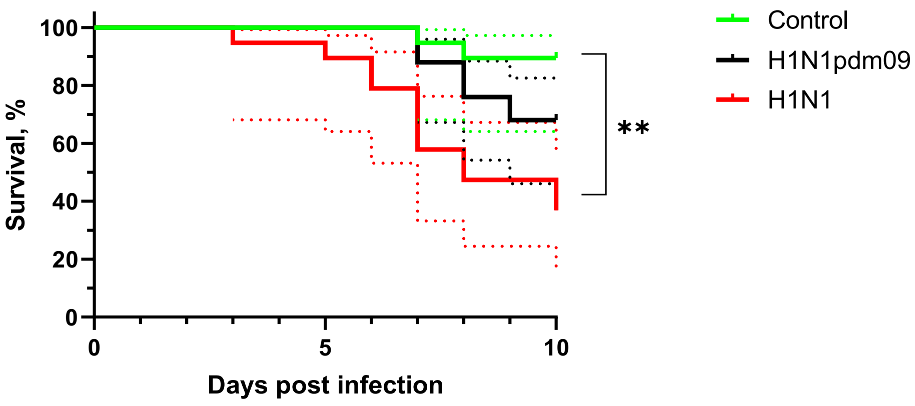

Figure S1. Infection with H1N1 resulted in approximately 50% mortality rate in pregnant mice. Significant differences were shown between H1N1wsn (red) and control (green). Data are presented as Kaplan–Meier survival curves with the 95% CIs of the survival estimates. **p < 0.01 with Bonferroni correction for multiple comparation.

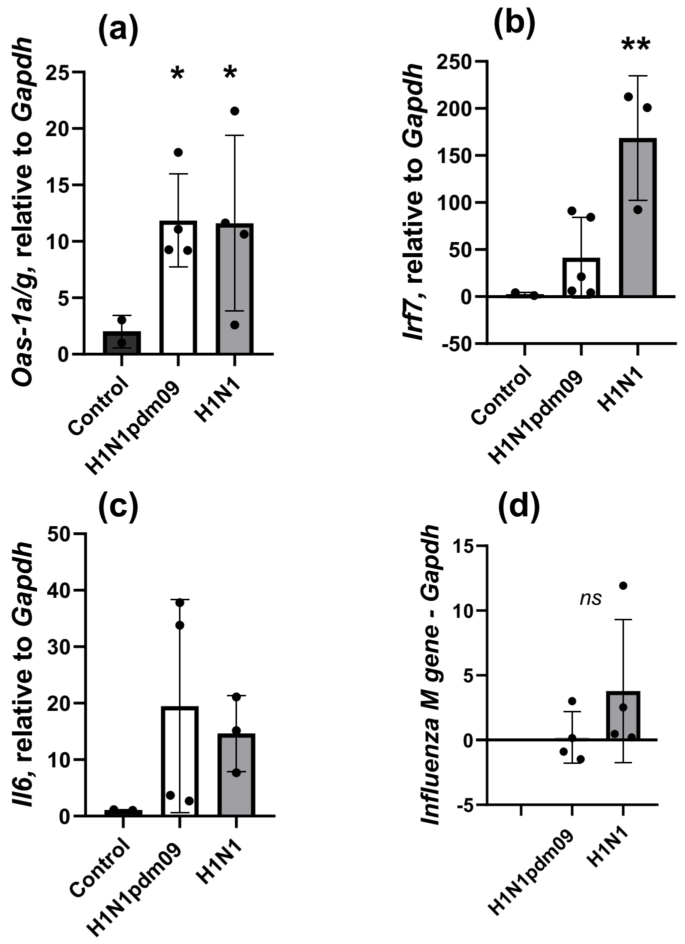

Figure S2. Infection with IAV caused the expression of *Oas-1a/g* **(a)**, *Irf7* **(b)**, and *Il6* **(c)** in lung homogenates of mothers after childbirth. RNA of M-protein was detected in lung homogenates **(d)**. Data are presented as mean ± SD. *p < 0.05; **p<0.01. Statistical analysis was performed using one-way ANOVA with Dunnett's multiple comparisons test (a-c) or t-test (d).

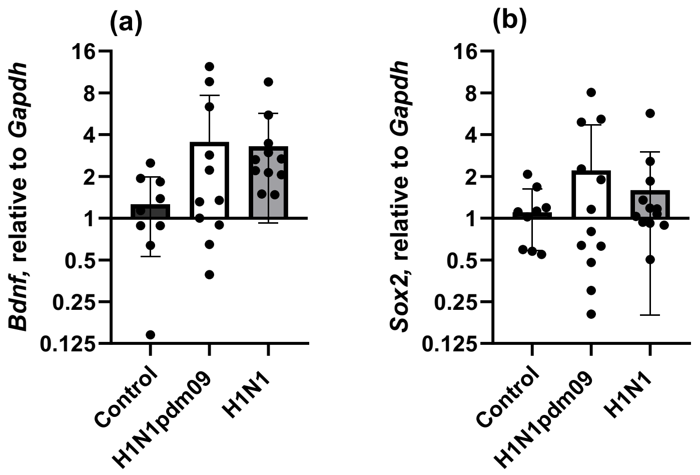

Figure S3. Expression of neurotropic factors in hippocampal tissues of newborn pups (p0) whose mothers were infected with H1N1pdm09 or H1N1wsn. The expression of *Bdnf* and *Sox-2* in hippocampi was at the basal level. Data are presented as mean ± SD. Statistical analysis was performed using one-way ANOVA.

Table S1. Primers and probes

Gene	mRNA	Sequence (5’-3’)
Bdnf	*NM_007540.4*<br>*NM_001048139.1*<br>*NM_001048141.1*<br>*NM_001048142.1*<br>*NM_001285416.1*<br>*NM_001285417.1*<br>*NM_001285418.1*<br>*NM_001285419.1*<br>*NM_001285420.1*<br>*NM_001285421.1*<br>*NM_001285422.1*<br>*NM_001316310.1*	GCGCCCATGAAAGAAGTAAA<br>ATGACGTGCTCAAAAGTGTC<br>HEX-AGGTTCGAGAGGTCTGACGACGACAT-BHQ2
Ifnb	*NM_010510.1*	ATGAGTGGTGGTTGCAGGC<br>TGACCTTTCAAATGCAGTAGATTCAC<br>Cy5-AAGCATCAGAGGCGGACTCTGGGAC-BHQ3
Il-1β	*NM_008361.4*	TGACGTTCCCATTAGACAACT<br>AGCTCATGGAGAATATCACTTG<br>HEX-ACTACAGGCTCCGAGATGAACAACA-BHQ2
Il-6	*NM_031168.2*<br>*NM_001314054.1*	TGATGGATGCTACCAAACTGGA<br>CTGAAGGACTCTGGCTTTGTCT<br>ROX-CTTCTGGAGTACCATAGCTACCTGGAGTA-BHQ2
iNos	*NM_010927.4*<br>*NM_001313921.1*<br>*NM_001313922.1*	TTGAAGCCCCGCTACTACTC<br>AAGCCACTGACACTTCGCAC<br>ROX-CACGGCCACAGTGAGGTGAACCT-BHQ2
Irf-7	*NM_016850.3*<br>*NM_001252600.1*<br>*NM_001252601.1*	CCTGGAAGCATTTCGGTCGT<br>CTCTTCGCTCTCTTCGCTCA<br>Cy5.5-CCACCTAGTGGAGTTAACCTGCCA-BHQ1
Oas1a/g	*NM_145211.2*<br>*NM_011852.3*<br>*NM_001424703.1*<br>*NM_001424704.1*<br>*NM_001424705.1*	GATGTGCCGACGGTGGT<br>TGGACAGGAGTCAAACATGGC<br>HEX-CACCTGGCATCAGACTCCGTGCTTCT-BHQ2

	NM_001424706.1<br>NM_001424707.1	
Sox-2	NM_011443.4	ACTTTTGTCCGAGACCGAGA<br>TCCGGGAAGCGTGTACTTAT<br>Cy5-GAAAACCAAGACGCTCATGAAGAAGG-BHQ3
Tnf-α	NM_013693.3<br>NM_001278601.1	CCACCACGCTCTTCTGTCTAC<br>GAGGGTCTGGGCCATAGAACT<br>Cy5-AAGTTCCCAAATGGCCTCCCTCTCATCAG-BHQ3
Gapdh	NM_001289726.2<br>NM_008084.4<br>NM_001411840.1<br>NM_001411841.1<br>NM_001411842.1<br>NM_001411843.1<br>NM_001411844.1<br>NM_001411845.1	AATGGTGAAGGTCGGTGTG<br>ACAAGCTTCCCATTCTCGG<br>FAM-TTGACTGTGCCGTTGAATTTGCCG-BHQ1

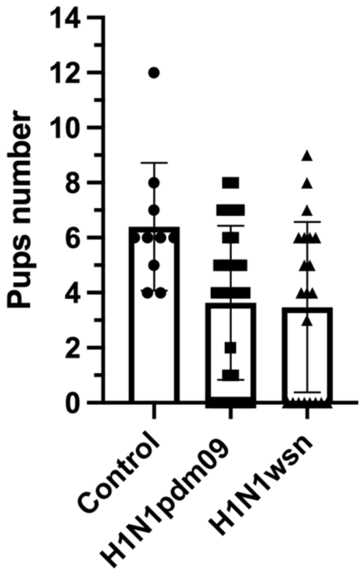

Figure S4. Pregnant dams were infected with H1N1pdm09 or H1N1wsn, and the average number of neonates was counted. Statistical analysis was performed using one-way ANOVA.

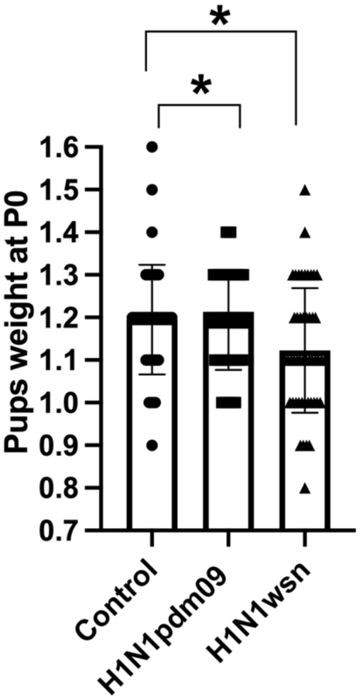

Figure S5. Newborn pups from IAV-infected mothers had reduced weight. Pregnant dams were infected with H1N1pdm09 or H1N1wsn, and the average weight of neonates was counted. The pups' number in groups: control - 42, H1N1pdm09 – 55; H1N1wsn – 49. Data are presented as mean ± SD. Statistical analysis was performed using one-way ANOVA with Tukey's test for multiple comparisons (* – $p < 0.05$).
